# A novel human anti-VCAM-1 monoclonal antibody ameliorates airway inflammation and remodelling

**DOI:** 10.1111/jcmm.12102

**Published:** 2013-07-16

**Authors:** Jae-Hyun Lee, Jung-Ho Sohn, Su Yeon Ryu, Chein-Soo Hong, Kyung D Moon, Jung-Won Park

**Affiliations:** a Division of Allergy and Immunology, Department of Internal Medicine, Yonsei University College of MedicineSeoul, Korea; b Institute of Allergy, Yonsei University College of MedicineSeoul, Korea; c Hanwha Chemical R&D CenterDaejeon, Korea

**Keywords:** VCAM-1, monoclonal antibody, allergic inflammation, asthma, cell adhesion molecule, anti-inflammatory effect

## Abstract

Asthma is a chronic inflammatory disease induced by Type 2 helper T cells and eosinophils. Vascular cell adhesion molecule-1 (VCAM-1) has been implicated in recruiting eosinophils and lymphocytes to pathological sites in asthma as a regulatory receptor. Accordingly, monoclonal antibody (mAb) against VCAM-1 may attenuate allergic inflammation and pathophysiological features of asthma. We attempted to evaluate whether a recently developed human anti-VCAM-1 mAb can inhibit the pathophysiological features of asthma in a murine asthma model induced by ovalbumin (OVA). Leucocyte adhesion inhibition assay was performed to evaluate the *in vitro* blocking activity of human anti-VCAM-1 mAb. OVA-sensitized BALB/c mice were treated with human anti-VCAM-1 mAb or isotype control Ab before intranasal OVA challenge. We evaluated airway hyperresponsiveness (AHR) and bronchoalveolar lavage fluid analysis, measured inflammatory cytokines and examined histopathological features. The human anti-VCAM-1 mAb bound to human and mouse VCAM-1 molecules and inhibited adhesion of human leucocytes *in vitro*. AHR and inflammatory cell counts in bronchoalveolar lavage fluid were reduced in mice treated with human anti-VCAM-1 mAb as compared with a control Ab. The levels of interleukin (IL)-5 and IL-13, as well as transforming growth factor-β, in lung tissue were decreased in treated mice. Human anti-VCAM-1 mAb reduced goblet cell hyperplasia and peribronchial fibrosis. *In vivo* VCAM-1 expression decreased in the treated group. In conclusion, human anti-VCAM-1 mAb attenuated allergic inflammation and the pathophysiological features of asthma in OVA-induced murine asthma model. The results suggested that human anti-VCAM-1 mAb could potentially be used as an additional anti-asthma therapeutic medicine.

## Introduction

Asthma is a chronic respiratory disease and its prevalence is increasing rapidly worldwide. The characteristic pathophysiological features of asthma are chronic eosinophilic inflammation of airways, airway hyperresponsiveness (AHR) and reversible airflow obstruction [1]. Many cytokines play important roles in chronic allergic inflammation development, among which interleukin (IL)-4, IL-5 and IL-13 [cytokines from type 2 helper T cells (Th2)] are key mediators thereof [2]. In particular, IL-5 recruits eosinophils to inflamed sites and prolongs the lifespan thereof. Accordingly, assessing the number of eosinophils in induced sputa of asthma patients may be clinically relevant to diagnosing and monitoring treatment response [3, 4].

The first step in the recruiting of inflammatory cells in asthma development is cellular adhesion to vascular endothelial surfaces, and various cell adhesion molecules are known to play critical roles in adhering leucocytes to endothelial cells. Vascular cell adhesion molecule-1 (VCAM-1), known as CD106, is the ligand of very late antigen-4 (VLA-4), which is expressed along the surface of the plasma membrane of eosinophils. Thus, VCAM-1 may play an important role in the development of eosinophilic inflammation [5]. Adhesion molecules, including intercellular cell adhesion molecule-1 (ICAM-1) and VCAM-1, are primarily expressed on the surface of vascular endothelial cells. Once inflammation begins, their extracellular expression increases in endothelial cells *via* inflammatory mediators [6].

Allergic airway diseases such as asthma and allergic rhinitis are characterized by Th2 inflammation. IL-4 and IL-13 potentiate VCAM-1 expression in vascular endothelial cells, accelerating eosinophilic inflammation [7, 8]. In regulating VCAM-1 expression, nuclear factor-kappaB (NF-κB) is important and can be restricted by Poly [ADP-ribose] polymerase 1 (PARP-1) [9]. Medications that inhibit cysteinyl leukotriene-1 receptor such as montelukast can affect the adherence of eosinophils to VCAM-1 [10].

In ovalbumin (OVA)-induced murine models of acute asthma, systemically administrated rat anti-murine VCAM-1 antibody (Ab) and rat anti-murine VLA-4 Ab have been shown to reduce eosinophil infiltration into tracheal tissue [11]. Thus, VCAM-1 could be a novel therapeutic target for several diseases characterized by eosinophilic inflammation such as asthma, allergic rhinitis and eosinophilic bronchitis. In atopic dermatitis mouse models, VCAM-1 blockade was reported to delay disease onset and its severity [12]. In addition to these allergic diseases, LV remodelling after various heart diseases has also been shown to be associated with VCAM-1 expression, and its blockade could be important to reducing myocardial fibrosis [13].

Inhaled corticosteroids as potent anti-inflammatory drugs have been established as the primary treatments for persistent allergic asthma. Recently, several biological agents, including anti-immunoglobulin E (IgE) monoclonal Ab (mAb) [14], anti-IL-13 mAb [15] and anti-IL-5 mAb [16], have been developed for difficult-to-treat or severe asthma. As mentioned in these previous studies, one potential pitfall of these biological agents is their safety. In this regard, human or humanized isoform antibodies rather than chimeric forms should be considered for development to minimize unexpected auto-immune reactions in humans.

In this study, we tested whether a novel monoclonal antibody designed to bind human VCAM-1 molecule attenuated allergic inflammation and ameliorated the pathophysiological features of asthma in an OVA-induced murine model.

## Materials and methods

### Reagents and animals

We used human anti-VCAM-1 mAb (HD101) (Hanwha Chemical, Daegeon, Korea) that bound both human and mouse VCAM-1. HD101 was designed to bind to domains 1 and 2 of VCAM-1, specifically, has and comprises an immunoglobulin G4 (IgG4) backbone (molecular weight 150 kD). Female 6- to 8-week-old BALB/c mice (Orient, Daegeon, Korea) were used for all experiments. All mice were kept under specific pathogen-free conditions, according to the standards of the American Association for the Accreditation of Laboratory Animal Care-approved facilities. All experiments described in this study were approved by the Animal Research Ethics Board of Yonsei University (Seoul, Korea).

### Cross-reactivity ELISA assay

A 96-well plate was coated with recombinant human VCAM-1/Fc (862-VC, R&D systems, Minneapolis, MN, USA) or mouse VCAM-1/Fc (643-VM, R&D systems) at 4°C overnight. The plate was then washed with PBS and blocked with 1% bovine serum albumin (BSA) in PBS at 37°C for 2 hrs. Thereafter, human anti-VCAM-1 mAb was added at 37°C for 2 hrs. The binding affinity of human anti-VCAM-1 mAb to coated VCAM-1 molecule was observed with horseradish peroxidase (HRP)-conjugated anti-F(ab')_2_ Ab using 3,3,5,5-tetramethylbenzidine (TMB) colorigenic substrate. To stop enzyme–substrate reaction, 1 N H_2_SO_4_ was added. Absorbance [optical density (OD) values] was measured at 450–650 nm.

### Adhesion inhibition assays for recombinant VCAM-1 and HUVEC expressing VCAM-1

Each well of a 96-well plate (446612, Nunc, Roskilde, Denmark) was coated with 100 μl of recombinant human VCAM-1 (2 μg/ml for U937 and CD4^+^ T cell assay, 5 μg/ml for EoL-1 cell assay, 809-VR, R&D systems) at 4°C for 16 hrs. The plate was then blocked with 1% BSA in PBS for 2 hrs at room temperature (RT). Then, human anti-VCAM-1 mAb was added to the VCAM-1-coated wells for antigen binding for 1 hr at RT. Meanwhile, human leucocytes—U937 cells (CRL-1593.2; ATCC, Manassas, VA, USA), EoL-1 cells (94042252; ECACC, Salisbury, UK) or CD4^+^T cells (isolated from human peripheral blood mononuclear cells, CC-2702, Lonza, Basel, Swiss)—were stained with 5 μM carboxyfluorescein diacetate succinimidyl ester (CFSE) (C34554; Invitrogen, Carlsbad, CA, USA). Fluorescence-labelled cells were incubated at 37°C for 15 or 30 min. to allow cells to interact with coated recombinant VCAM-1. Non-adherent cells were removed by centrifuging the sealed plate at 200 × g for 5 min. inverted, and 150 μl of cell lysis buffer (50 mM Tris-HCl, pH 8.5, 0.1% SDS) was added to each well for 10 min. to lyse the bound cells. Subsequently, fluorescence intensity was measured at 485–530 nm emission wavelengths using a fluorometer (GeminiX, Molecular Devices, Sunnyvale, CA, USA). Values represent the means of triplicate measurements for each condition. Finally, the reduction in fluorescence intensity in samples was analysed as the inhibition per cent compared to the isotype antibody-treated group.

Human umbilical vein endothelial cells (HUVECs, Lonza) were plated on a 96-well plate at a density of 2 × 10^4^ cells per well, and cultured in EGM-2 media (Lonza) for 3–4 days according to the manufacturer's instructions. HUVEC cells of passages 1–4 were stimulated with 20 ng/ml of human tumour necrosis factor (TNF)-α (Abcam, Cambridge, UK) for 24 hrs to express VCAM-1 molecules. Each well was washed twice with 200 μl of EGM-2 medium, and human anti-VCAM-1 mAb was added. Prepared fluorescent-labelled U937 cells were then added to interact with VCAM-1 expressed on HUVEC surfaces. Serial experimental steps were performed as mentioned above.

### VCAM-1 internalization assay

Tumour necrosis factor-α–primed HUVECs were prepared as above. Human anti-VCAM-1 mAb (10 μg/ml) was then added and cells were incubated at 4°C. After fixation on ice, the mixture was incubated at 37°C for 10 or 60 min. for internalization of VCAM-1/mAb complexes, and cells were then washed with acidic PBS (pH 2.5) to remove VCAM-1/mAb complexes from cell surfaces. FITC-conjugated goat anti-human IgG Ab (Sigma-Aldrich, St. Louis, MO, USA) was added to detect human anti-VCAM-1 mAb in permeabilized cells. Internalization of VCAM-1/mAb complexes was analysed by flow cytometry and mean fluorescence intensity (MFI) values were compared.

### Murine model of OVA induced acute and chronic asthma

To induce acute asthma in mice, on experimental days 1 and 14, mice were systemically sensitized with 10 μg of OVA (Sigma-Aldrich) mixed with 1% aluminium hydroxide (Resorptar, Indergen, New York, NY, USA) *via* intraperitoneal injection. One week after the second sensitization, mice were challenged with three intranasal inoculations of 1% OVA in 30 μl of PBS under inhalation anaesthesia. Five percent isoflurane was used for anaesthesia, it was well known that isoflurane do not promote inflammation in the lung tissue [17, 18]. Antibody treatment [100 μg/mouse (about 4 mg/kg), intravenously] was administered once, 1 day prior to OVA challenge. To induce chronic asthma in mice, as described above, mice were sensitized twice. One week after sensitization, intranasal inoculations were administered twice weekly for 4 weeks. A total of four intravenous (IV) Ab treatments (100 μg/mouse for each time) were administered at weekly intervals, beginning 1 day before OVA challenge.

### Measurement of AHR

Forty-eight hours after the last OVA challenge, AHR to methacholine (MCh, Sigma-Aldrich) was measured. In each mouse, a tracheostomy was performed and an 18-gauge steel tube was inserted for ventilation with a small animal ventilator (Flexivent 5.1®, Scireq, Montreal, QC, Canada) at a respiration rate of 160 breaths/min. with a 10 ml/kg tidal volume under appropriate anaesthesia. A positive end expiratory pressure of 3 cm H_2_O was applied. Mice were challenged with saline aerosol at baseline followed by serially increasing concentrations of MCh (3.1, 6.25, 12.5, 25.0 and 50.0 mg/ml). Each aerosol was delivered *via* the nebulizer of the Flexivent® system using a full inspiration manoeuvre (respiration rate 60 breaths/min., tidal volume 25 ml/kg) for 12 sec. (10 full inspirations).

### Analysis of bronchoalveolar lavage fluid

Bronchoalveolar lavage (BAL) in each mouse was performed with 1 ml of Hank's balanced salt solution (HBSS) to collect BAL fluid *via* tracheostomy tube. The total inflammatory cell count was measured by a hemocytometer. The BAL fluid was then centrifuged at 1.5 × *g* for 3 min. at 4°C to collect supernatants. After removing the supernatant, cell pellets were re-suspended in HBSS. A BAL cell smear was made by cytocentrifugation (cytospin3, Thermo, Waltham, MA, USA) at 200 × *g* for 3 min. at RT. All cell smear slides were stained using the Diff Quick staining method. Differential inflammatory cell counts included at least 200 neutrophils, eosinophils, lymphocytes or macrophages.

### Preparation of lung homogenate

After collecting BAL fluid, the right lung was resected from the bronchial tree and weighed. Then, it was homogenized using a tissue homogenizer (Biospec Products, Bartlesville, OK, USA) in 3 ml of lysis buffer containing 0.5% Triton X-100, 150 mM NaCl, 15 mM Tris, 1 mM CaCl_2_, 1 mM MgCl_2_ and protease inhibitor solution (Sigma-Aldrich) at pH 7.4. After incubating for 30 min. on ice, homogenates were centrifuged at 14,000 × *g* for 10 min. Supernatants were harvested and passed through a 0.45–micron filter (Gelman Sciences, Ann Arbor, MI, USA). The final preparations were stored at −20°C for cytokine measurements.

### Cytokine level measurement

We used ELISA kits (R&D Systems) to measure IL-5 (detection limit: 31.2 pg/ml), IL-13 (detection limit: 62.5 pg/ml), interferon (IFN)-γ (detection limit: 31.2 pg/ml) and transforming growth factor (TGF)-β (detection limit: 31.2 pg/ml) in the lung homogenates.

### Histopathological examination

After collecting BAL fluid, the left lung was filled with 10% formalin solution, embedded in paraffin and cut into 3-μm-thick sections. We utilized haematoxylin and eosin staining for general examination, periodic acid-Schiff staining (PAS) to measure goblet cell hyperplasia and Masson's trichrome staining to measure fibrosis. In addition to light microscopic examination, quantification analysis was conducted using software (Metamorph®, Molecular Devices). Briefly, PAS-stained slides were placed under a light microscope at 200× magnification. Circling two bronchi along the basement membrane on each slide, the number of goblet cells in selected bronchi was counted. Finally, goblet cell numbers per each micrometre of basement membrane were calculated and analysed statistically. Fibrosis area was measured by colour pixel count over pre-set threshold colour for the entire field containing several bronchovascular bundles.

### Immunohistochemical examination

Slides were prepared as described in ‘Histopathological examination’, deparaffinized three times with xylene and washed in ethanol. Hydrogen peroxide (3% in methanol) was used to inactivate endogenous peroxidases. Slides were incubated with anti-VCAM-1 Ab (Millipore, Billerica, MA, USA) for 1 hr at RT, followed by 4°C for 24 hrs. After washing in PBS, slides were treated with secondary antibody (DakoCytomation, Carpentaria, CA, USA) and incubated for 20 min. at room temperature. Diaminobenzidine (DAB) was developed for 5 min. Slides were counterstained with haematoxylin and mounted using mounting medium.

### Statistical analysis

Statistical differences among mean values were analysed using one-way anova. Differences in AHR were analysed using repeated measures anova with a post-hoc Bonferroni test. In all cases, *P* < 0.05 was considered statistically significant. We used SPSS statistical software version 12.0 (SPSS Inc., Chicago, IL, USA) to conduct all analyses.

## Results

### Cross-reactivity of human anti-VCAM-1 mAb against murine VCAM-1

The affinities of human anti-VCAM-1 mAb for human and mouse VCAM-1 were evaluated by ELISA method. The antibody bound both human and mouse VCAM-1. Although the antibody was designed to bind human VCAM-1, it had good affinity for human VCAM-1 at concentrations over 10 ng/ml (OD 0.187 ± 0.040 at 10 ng/ml, OD 0.880 ± 0.132 at 100 ng/ml) and it also bound mouse VCAM-1 at 100 ng/ml (OD 0.170 ± 0.009).

### Leucocyte adhesion inhibition to recombinant human VCAM-1 and HUVECs expressing VCAM-1

**Fig. 1:**
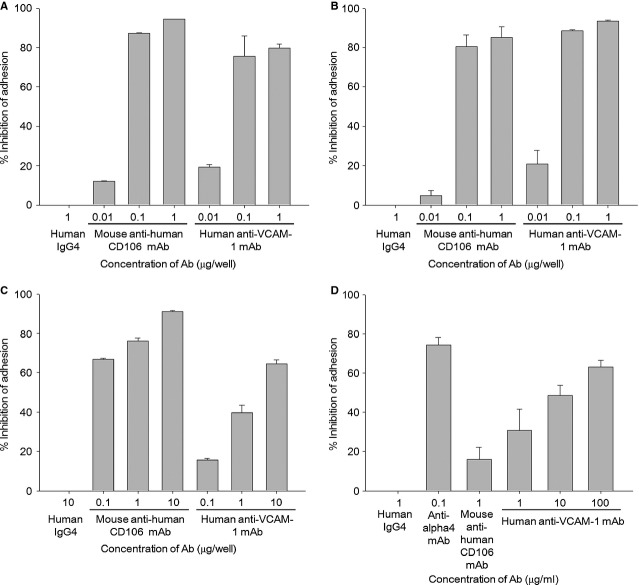
Leucocyte adhesion inhibition assay. Human anti-vascular cell adhesion molecule (VCAM)-1 mAb effectively inhibited leucocytes from binding to recombinant VCAM-1. U937 cell (**A**) and EoL-1 cell (**B**) adhesion was inhibited with a 0.1 μg/well concentration. In CD4^+^ T cell (**C**), 64.5 ± 3.3% inhibition was obtained at 10 μg/well concentration. Human anti-VCAM-1 mAb effectively inhibited U937 cell from binding to a VCAM-1 expressing HUVEC mono-layer in a dose-dependent manner (**D**). All data showed as mean ± SEM of three independent assays.

Human anti-VCAM-1 mAb inhibited U937, EoL-1 and CD4^+^ T cells from adhering to recombinant human VCAM-1 molecules. Marked interference was observed at concentrations over 0.1 μg/well (Fig. 1). U937 and EoL-1 cell adhesion to recombinant human VCAM-1 were effectively blocked at 0.1 μg of human anti-VCAM-1 mAb/well (75.5 ± 14.9% for U937, 88.4 ± 0.9% for EoL-1; Fig. 1A and B). The adhesion inhibition assay for CD4^+^ T cell showed up to 64.5 ± 3.3% inhibition (at 10 μg/well) in a dose-dependent manner (Fig. 1C). In addition, human anti-VCAM-1 mAb inhibited U937 cells from adhering to a HUVEC mono-layer primed with TNF-α to express VCAM-1 in a dose-dependent manner. The maximum inhibition was 63.1 ± 5.9% at 100 μg/ml (Fig. 1D).

### Internalization of VCAM-1 into cytosol

**Fig. 2:**
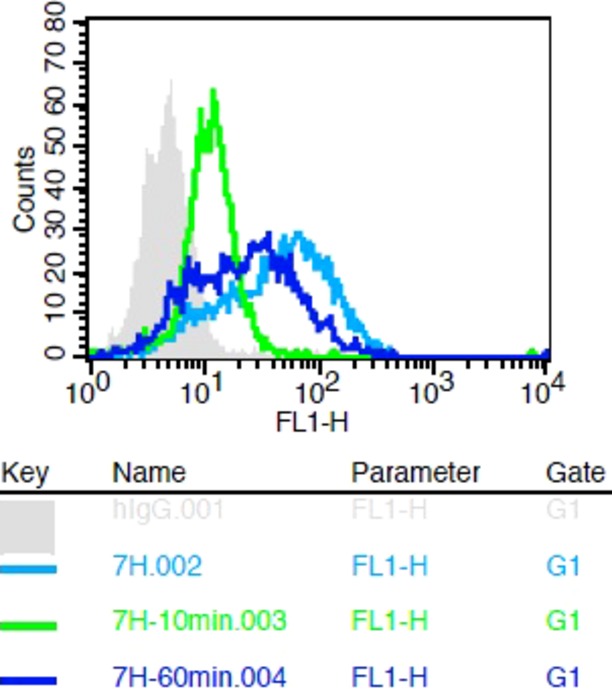
Internalization assay. Human anti-vascular cell adhesion molecule (VCAM)-1 mAb treatment induced internalization of VCAM-1 molecules. Internalization of VCAM-1/mAb complexes was analysed by flow cytometry (two independent assays were performed). The grey area represents isotype control (MFI 5.2). Each coloured lines represent the following: sky blue line, total VCAM-1 expression (MFI 62.4); green line, for 10 min. (MFI 14.7); and blue line, for 60 min. (MFI 32.8).

Tumour necrosis factor-α–primed HUVECs expressed VCAM-1 molecules on their surface. The addition of human anti-VCAM-1 mAb induced internalization of VCAM-1 into cytosol. The MFI of total expression was 62.4. The MFI values of internalized VCAM-1 were 14.7 at 10 min. and 32.8 at 60 min. after human anti-VCAM-1 mAb treatment (Fig. 2).

### The effects of human anti-VCAM-1 mAb on pathophysiological features in acute asthma murine model

**Fig. 3:**
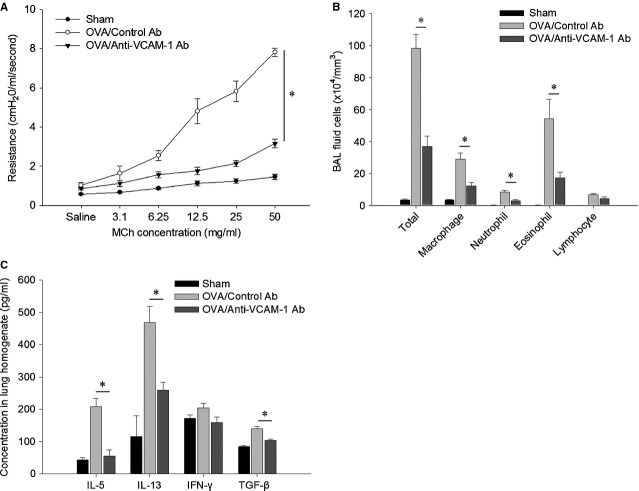
Changes in physiological features and cytokine profile in an acute asthma model. Human anti-vascular cell adhesion molecule (VCAM)-1 mAb treatment attenuated airway hyperresponsiveness induced by MCh provocation (**A**). Inflammatory cells were decreased in bronchoalveolar lavage fluid significantly (**B**). All cytokines were measured from homogenates of the right lung of each mouse. IL-5, IL-13 and transforming growth factor-β levels in the lung homogenates were significantly reduced in the treated group; however, IFN-γ level showed no difference (**C**). In each group, five mice were allocated. All data showed as mean ± SEM. *P < 0.05 between OVA/control Ab and OVA/human anti-VCAM-1 mAb group.

We evaluated the anti-inflammatory effects of IV treatment with human anti-VCAM-1 mAb on the pathophysiological features of asthma *via* an OVA-induced acute asthma model. Administration of 100 μg of IV human anti-VCAM-1 mAb significantly reduced MCh-induced AHR [*P* < 0.001; resistance at 25 mg/ml, 5.83 ± 1.16 (OVA/control Ab group) *versus* 2.14 ± 0.34 cm H_2_O (OVA/human anti-VCAM-1 mAb group); at 50 mg/ml, 7.82 ± 0.47 *versus* 3.16 ± 0.51 cm H_2_O; Fig. 3A].

Bronchoalveolar lavage fluid analysis showed significantly reduced eosinophilic and neutrophilic inflammation and fewer macrophages as a result of IV human anti-VCAM-1 mAb [total inflammatory cell number 98.4 ± 19.4 (OVA/control Ab group) *versus* 36.8 ± 14.9 × 10^4^/mm^3^ (OVA/human anti-VCAM-1 mAb group), *P* < 0.001; macrophage, 29.0 ± 8.4 *versus* 12.1 ± 4.9 × 10^4^/mm^3^, *P* = 0.001; neutrophil, 8.3 ± 2.8 *versus* 3.0 ± 1.6 × 10^4^/mm^3^, *P* = 0.002; eosinophil, 54.3 ± 26.9 *versus* 17.3 ± 7.7 × 10^4^/mm^3^, *P* = 0.009; Fig. 3B].

Interleukin-5, IL-13 and TGF-β levels in lung homogenate were significantly lower in the human anti-VCAM-1 mAb–treated group [IL-5 level 208.8 ± 56.7 (OVA/control Ab group) *versus* 55.0 ± 41.7 pg/ml (OVA/human anti-VCAM-1 mAb group), *P* < 0.001; IL-13, 428.6 ± 143.6 *versus* 259.0 ± 53.5 pg/ml, *P* = 0.042; TGF-β, 139.8 ± 14.6 *versus* 103.4 ± 9.8 pg/ml, *P* = 0.001], but IFN-γ levels did not differ between groups (Fig. 3C).

**Fig. 4:**
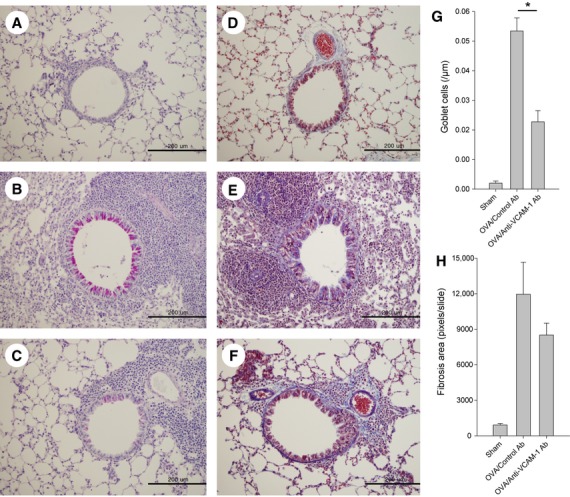
Changes in histopathological features in an acute asthma model. IV administration of human anti-vascular cell adhesion molecule (VCAM)-1 mAb attenuated peribronchial and perivascular inflammation as well as mucus metaplasia of respiratory epithelium, compared with the control Ab-treated group (**A**: sham, **B**: OVA/control Ab, **C**: OVA/human anti-VCAM-1 mAb; PAS stain; **G**: goblet cell count). However, lung fibrosis findings were not remarkable (**D**: sham, **E**: OVA/control Ab, **F**: OVA/human anti-VCAM-1 mAb; Masson's trichrome stain; **H**: calculated fibrosis area). In each group, five mice were allocated. All data showed as mean ± SEM. *P < 0.05 between OVA/control Ab and OVA/human anti-VCAM-1 mAb group.

On histopathological examination, we observed remarkable decreases in peribronchial and perivascular inflammatory cell infiltration. Goblet cell hyperplasia in the bronchial epithelia was also markedly decreased in mice treated with human anti-VCAM-1 mAb (Fig. 4A–C). Quantification analysis also revealed a decreased number of goblet cells in the human anti-VCAM-1 mAb–treated group [goblet cell number 0.053 ± 0.021 cells/μm (OVA/control Ab group) *versus* 0.039 ± 0.015 cells/μm (OVA/human anti-VCAM-1 mAb group), *P* = 0.043; Fig. 4G]. There was no significant difference in peribronchial fibrosis (Fig. 4D–F and H).

### The effects of human anti-VCAM-1 mAb on pathophysiological features in chronic asthma murine model

**Fig. 5:**
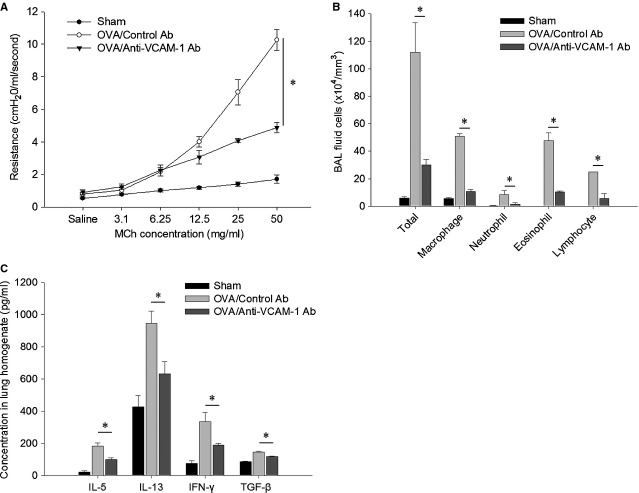
Changes in physiological feature and cytokine profile in a chronic asthma model. Human anti-vascular cell adhesion molecule (VCAM)-1 mAb treatment attenuated airway hyperresponsiveness induced by MCh provocation (**A**). Inflammatory cells were decreased in bronchoalveolar lavage fluid significantly (**B**). All cytokines were measured from homogenates of the right lung of each mouse. IL-5, IL-13, IFN-γ and transforming growth factor-β levels in the lung homogenates were significantly reduced in the treated group (**C**). In each group, five mice were allocated. All data showed as mean ± SEM. *P < 0.05 between OVA/control Ab and OVA/human anti-VCAM-1 mAb group.

In the chronic asthma mouse model, we observed similar anti-inflammatory and anti-asthma effects for IV human anti-VCAM-1 mAb. Four treatments (100 μg each) significantly reduced MCh-induced AHR [*P* < 0.001; resistance at 25.0 mg/ml 7.06 ± 1.72 (OVA/control Ab group) *versus* 4.09 ± 0.26 cm H_2_O (OVA/human anti-VCAM-1 mAb group); at 50.0 mg/ml, 10.26 ± 1.42 *versus* 4.88 ± 0.71 cm H_2_O; Fig. 5A].

In the BAL fluid analysis, total inflammatory cells, eosinophils, neutrophils, macrophages and lymphocytes were significantly decreased in the human anti-VCAM-1 mAb–treated group [total inflammatory cell number 120.4 ± 29.2 (OVA/control Ab group) *versus* 29.6 ± 9.9 × 10^4^/mm^3^ (OVA/human anti-VCAM-1 mAb group), *P* < 0.001; macrophage, 46.0 ± 14.8 *versus* 11.2 ± 2.5 × 10^4^/mm^3^, *P* < 0.001; neutrophil, 9.4 ± 4.1 *versus* 2.0 ± 1.3 × 10^4^/mm^3^, *P* = 0.001; eosinophil, 45.6 ± 17.6 *versus* 9.3 ± 4.1 × 10^4^/mm^3^, *P* < 0.001; lymphocyte, 19.4 ± 10.5 *versus* 7.2 ± 4.8 × 10^4^/mm^3^, *P* = 0.033; Fig. 5B].

Interleukin-5, IL-13, TGF-β and IFN-γ levels were decreased in lung homogenates from the treated group [IL-5 level, 182.6 ± 42.9 (OVA/control Ab group) *versus* 98.6 ± 26.2 pg/ml (OVA/human anti-VCAM-1 mAb group), *P* = 0.003; IL-13, 944.4 ± 169.9 *versus* 631.0 ± 173.3 pg/ml, *P* = 0.029; IFN-γ, 335.4 ± 121.4 *versus* 21.8 ± 7.0 pg/ml, *P* = 0.023; TGF-β, 144.4 ± 14.0 *versus* 116.6 ± 9.0 pg/ml, *P* = 0.003; Fig. 5C].

**Fig. 6:**
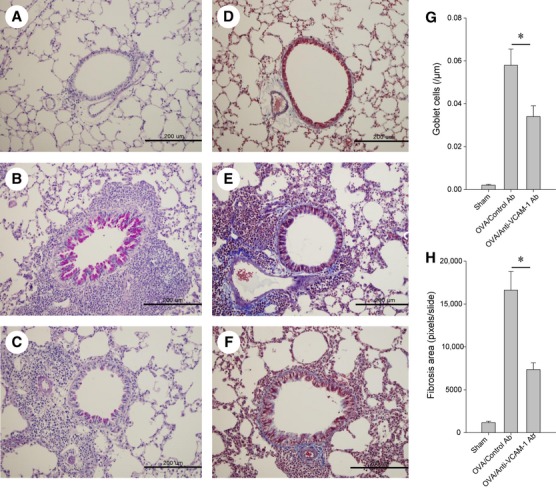
Changes in histopathological features in a chronic asthma model. Four human anti-vascular cell adhesion molecule (VCAM)-1 mAb treatments attenuated peribronchial and perivascular inflammation and goblet cell hyperplasia of respiratory epithelium, compared with the control Ab-treated group (**A**: sham, **B**: OVA/control Ab, **C**: OVA/human anti-VCAM-1 mAb; PAS stain; **G**: goblet cell count). Moreover, peribronchial and perivascular fibrosis was reduced in the human anti-VCAM-1 mAb–treated group (**D**: sham, **E**: OVA/control Ab, **F**: OVA/human anti-VCAM-1 mAb; Masson's trichrome stain; **H**: calculated fibrosis area). In each group, five mice were allocated. All data showed as mean ± SEM. *P < 0.05 between OVA/control Ab and OVA/human anti-VCAM-1 mAb group.

Histopathological examination showed remarkably reduced peribronchial and perivascular inflammatory cell infiltration and goblet cell hyperplasia in the bronchial epithelia (Fig. 6A–C). Goblet cell quantification also revealed a significantly decreased cell number in the treated group [goblet cell number 0.042 ± 0.015 cells/μm (OVA/control Ab group) *versus* 0.026 ± 0.013 cells/μm (OVA/human anti-VCAM-1 mAb group), *P* = 0.041; Fig. 6G]. In terms of airway remodelling, perivascular and peribronchial fibrosis was also ameliorated in the treated group (Fig. 6D–F). Calculated fibrosis area was significantly decreased in the treated group [fibrosis area 16,616 ± 4374 (OVA/control Ab group) *versus* 7350 ± 1570 pixel/slide (OVA/human anti-VCAM-1 mAb, *P* = 0.002; Fig. 6H].

### The effects of human anti-VCAM-1 mAb on *in vivo* VCAM-1 expression

**Fig. 7:**
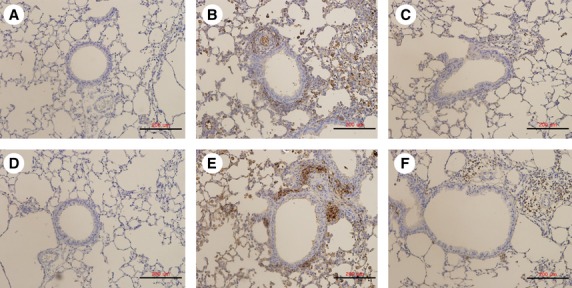
Changes of *in vivo* vascular cell adhesion molecule (VCAM)-1 expression pattern. IV administration of human anti-VCAM-1 mAb attenuated endothelial and epithelial VCAM-1 expression (brownish colour) in acute and chronic asthma mice (in two repetitive experiments) (**A**: sham of acute model, **B**: OVA/control Ab of acute model, **C**: OVA/human anti-VCAM-1 mAb of acute model, **D**: sham of chronic model, **E**: OVA/control Ab of chronic model, **F**: OVA/human anti-VCAM-1 mAb of chronic model).

Immunohistochemistry revealed increased VCAM-1 expression in the lung tissues of both acute and chronic OVA–induced asthma mice. In addition, we observed reduced expression of VCAM-1 in mice treated with human anti-VCAM-1 mAb in both the acute and chronic asthma models (Fig. 7).

## Discussion

In this study, we demonstrated that a novel human anti-VCAM-1 mAb exerted anti-inflammatory and anti-asthma effects. IV treatment with human anti-VCAM-1 mAb reduced allergic inflammation in BAL fluid, invasively measured AHR, inflammatory cell infiltration and goblet cell hyperplasia in the lung tissues of OVA-induced mouse models of acute and chronic asthma. Furthermore, mice with chronic asthma exhibited less fibrosis of lung tissues after treatment of human anti-VCAM-1 mAb. Therefore, we suggest that anti-VCAM-1 treatment could potentially be a new therapeutic modality in allergic airway diseases.

Th2 lymphocytes are known to orchestrate allergic asthma. Th2 cells produce cytokines including IL-4, IL-5 and IL-13 to influence B lymphocytes to differentiate into plasma cells and produce IgE. They also recruit eosinophils, the main effector cell, to inflammation sites [19]. At the initiation of allergic inflammation, few inflammatory mediators (such as histamine) and pro-inflammatory cytokines (such as TNF-α) are released at the primary site. These mediators stimulate endothelial cells to express cell adhesion molecules, such as ICAM-1 and VCAM-1 [20]. The cell adhesion molecules capture neutrophils, eosinophils and Th2 lymphocytes from the bloodstream and facilitate migration to the inflamed tissue. These recruited cells produce a large amount of cytokines and mediators, perpetuating the cycle [19]. In this study, HD101 effectively blocked interactions between VCAM-1 and VLA4 in *in vitro* adhesion assays in U937, EoL-1 and CD4^+^ T cells. In addition, HD101 showed adhesion blockage effect between VCAM-1-expressed HUVECs and U937 cells. Although we were unable to show *in vitro* reductions in transepithelial migration of these inflammatory effector cells, *in vivo* IV HD101 treatment reduced inflammatory cell infiltration in murine models of both acute and chronic asthma. Consequently, we speculate that human anti-VCAM-1 mAb blocks VCAM-1 molecules expressed on the surface of endothelial cells and inhibits recruitment of inflammatory effector cells and ameliorated allergic airway inflammation.

Our animal experiments revealed reduced AHRs in both acute and chronic models with IV HD101 treatment. These data were consistent with IL-13 levels in lung homogenates. Previously, IL-13 was known to be a key mediator of AHR development and goblet cell hyperplasia in an OVA-sensitized asthma model. Accordingly, blockade of IL-13 alone could dramatically reduce AHR [21]. Nevertheless, overexpression of IL-13 in lung tissue induced the development of AHR [22]. In our models, the decrement of IL-13 level was correlated with a reduction in goblet cell hyperplasia.

We also observed decreases in IL-5 levels in lung tissue and eosinophil counts in BAL fluid. Although the influence of eosinophils upon AHR is under debate [23–25], IL-5 is a crucial cytokine for AHR. IL-5 in allergic asthma pathogenesis primarily comes from Th2 lymphocytes and tissue-infiltrated eosinophils. In this regard, VCAM-1 blockade could reduce eosinophil influx and also decrease IL-5 levels in inflamed lung tissue. In a preliminary study, we evaluated the dose-responsive anti-inflammatory effects of human anti-VCAM-1 mAb and found that a dose of 100 μg/mouse (∼4 mg/kg) exerted the best responses in acute asthma mice.

In terms of airway resistance, the chronic model of this study showed greater increases in resistance than the acute model (with and without human anti-VCAM-1 mAb treatment). This finding could be explained by chronic allergen exposure and development of airway remodelling. Clinically, in some severe asthma patients with a long disease duration, airway remodelling, characterized by peribronchial and perivascular fibrosis, fixed airflow obstruction, and permanent loss of lung function, has been reported [26]. In this study, we evaluated fibrosis in both acute and chronic asthma models. The chronic model showed more severe peribronchial and perivascular fibrosis than the acute model as expected. Repetitive treatment of IV human anti-VCAM-1 mAb reduced fibrosis in the chronic model. However, one time injection of human anti-VCAM-1 mAb did not show effective fibrosis reduction in the acute model, although TGF-β level in lung tissues was decreased. Because tissue fibrosis results as a consequence of long-term exposure to high level of TGF-β [27], 5 days (from 1st OVA intranasal challenge to sacrifice) was too short of a period to assess fibrosis in lung tissue of acute asthma, even if TGF-β level was decreased. We thought evaluation of fibrosis using the chronic asthma model would be more reasonable and reliable in fibrosis analysis.

The main source of TGF-β stems from eosinophils recruited to the tissue. Accordingly, long-term VCAM-1 blockage may be protective against airway remodelling *via* decreased eosinophil influx to inflamed lung tissue, resulting in decreased TGF-β production. In this regard, we suggest human anti-VCAM-1 mAb treatment as an additional combination treatment option for severe asthma patients reflective of airway remodelling, despite receiving conventional asthma treatment including steroids (inhaled or systemic) [28].

Interestingly, we observed that human anti-VCAM-1 mAb treatment reduced *in vivo* expression of VCAM-1. This finding could be explained by our *in vitro* data from the internalization assay of VCAM-1 molecules in human anti-VCAM-1 mAb–treated HUVECs. From these results, VCAM-1 blockage therapy may exert one important additional effect of target molecule down-regulation by targeted therapy, although this phenomenon is well known in other monoclonal Abs [29] and adhesion molecules [30]. The mechanism of VCAM-1 internalization is known to be related with the clathrin-associated pathway [31].

In this study, we delivered the antibody intravenously. Further studies should test other delivery routes, such as intranasal or intratracheal inoculation, to evaluate their effects on airway epithelial cells and determine the dose reduction possibility using direct delivery to the target organ, because airway epithelial cells are important in the development and propagation of allergic inflammation. VCAM-1 and ICAM-1 expression is known to be increased in the bronchial epithelium of asthma patients, and these cell adhesion molecules have been shown to be related to the release of eosinophil cationic protein [32].

A few studies have assessed soluble (not membrane bound) VCAM-1 in the blood. Soluble VCAM-1 was increased in the sputa [33] and sera [34, 35] of asthma patients. Soluble VCAM-1 could increase the chemotactic activity of eosinophils *via* Src and MAP kinase signalling [36], but the pathophysiological roles of soluble VCAM-1 are not clear. Further study is needed to determine how human anti-VCAM-1 mAb affects soluble VCAM-1 to reduce inflammation.

In this study, we demonstrated decreased inflammation in allergic asthma in murine model. For future studies, we suggest that human anti-VCAM-1 mAb may exert anti-inflammatory effects in other eosinophilic diseases, such as eosinophilic gastroenteritis, Churg-Strauss syndrome and idiopathic hypereosinophilic syndrome [37]. We also expect that human anti-VCAM-1 mAb may have therapeutic effects on conditions related to VCAM-1 overexpression, such as atherosclerosis [38].

In conclusion, we demonstrated that a novel human anti-VCAM-1 mAb inhibited leucocytes from adhering to VCAM-1 molecules *in vitro*. Furthermore, we observed anti-inflammatory effects for IV treatment of human anti-VCAM-1 mAb in acute and chronic asthma mouse models, as well as an anti-fibrosis effect in chronic asthma mice. Further research concerning specific mechanisms of VCAM-1 blockade is warranted. For clinical implementation, well-designed clinical trials are crucial.
